# Association of ocular, cardiovascular, morphometric and lifestyle parameters with retinal nerve fibre layer thickness

**DOI:** 10.1371/journal.pone.0197682

**Published:** 2018-05-22

**Authors:** Julia Lamparter, Irene Schmidtmann, Alexander K. Schuster, Angeliki Siouli, Joanna Wasielica-Poslednik, Alireza Mirshahi, René Höhn, Josef Unterrainer, Philipp S. Wild, Harald Binder, Karl Lackner, Manfred E. Beutel, Thomas Münzel, Norbert Pfeiffer, Esther M. Hoffmann

**Affiliations:** 1 Department of Ophthalmology, University Medical Center Mainz, Mainz, Germany; 2 Augenzentrum Leinfelden-Echterdingen, Leinfelden-Echterdingen, Germany; 3 Institute for Medical Biometry, Epidemiology, and Informatics (IMBEI) of the University Medical Center Mainz, Mainz, Germany; 4 Clinic for Ophthalmology, Klinikum Frankfurt Höchst, Frankfurt, Germany; 5 Dardenne Eye Hospital, Bonn, Germany; 6 Department of Ophthalmology, Inselspital, University Hospital Bern, Bern, Switzerland; 7 Medical Psychology and Medical Sociology, Faculty of Medicine, University of Freiburg, Freiburg, Germany; 8 Preventive Cardiology and Preventive Medicine / Center for Cardiology, University Medical Center Mainz, Mainz, Germany; 9 Center for Thrombosis and Hemostasis (CTH), University Medical Center Mainz, Mainz, Germany; 10 German Center for Cardiovascular Research (DZHK), partner site Rhine-Main, Mainz, Germany; 11 Institute for Clinical Chemistry and Laboratory Medicine, University Medical Center Mainz, Mainz, Germany; 12 Department of Psychosomatic Medicine and Psychotherapy, University Medical Center Mainz, Mainz, Germany; 13 Center for Cardiology I, University Medical Center Mainz, Mainz, Germany; University of Florida, UNITED STATES

## Abstract

**Background:**

Glaucoma is a neurodegenerative disease, leading to thinning of the retinal nerve fibre layer (RNFL). The exact influence of ocular, cardiovascular, morphometric, lifestyle and cognitive factors on RNFL thickness (RNFLT) is unknown and was analysed in a subgroup of the Gutenberg Health Study (GHS).

**Methods:**

Global peripapillary RNFLT was measured in 3224 eyes of 1973 subjects (49% female) using spectral-domain optical coherence tomography (SD-OCT). The association of age, sex, ocular, cardiovascular, morphometric, lifestyle and cognitive factors on RNFLT was analysed using Pearson correlation coefficient and fitting a linear mixed model.

**Results:**

In the univariable analysis highest correlations were found for axial length (r = -0.27), spherical equivalent (r = 0.24), and glaucoma (r = -0.15) (p<0.0001, respectively). Other significant correlations with RNFLT were found for age, sex, intraocular pressure, systemic hypertension and systolic blood pressure, previous eye surgery, cholesterol, homocysteine, history of coronary artery disease, history of myocardial infarction, apnoea, diabetes and alcohol intake, p<0.05, respectively. Body length, body weight, BMI, diastolic blood pressure, blood glucose, HbA1c, history of apoplexy, cognitive function, peripheral artery disease, tinnitus, migraine, nicotine intake, central corneal thickness, and pseudophakia were not significantly correlated with RNFLT. The regression model revealed a significant relationship between RNFLT and age in decades (p<0.02), spherical equivalent (p<0.0001), axial length (p<0.0001), glaucoma (p<0.0001), tinnitus (p = 0.04), apnoea (p = 0.047), homocysteine (p = 0.05) and alcohol intake >10g/d for women and >20g/d for men (p = 0.02). Glaucoma, apnoea, higher homocysteine, higher alcohol intake and higher axial length as well as age were related to decreased RNFLT while higher spherical equivalent or history for tinnitus were related to thicker RNFL.

**Conclusion:**

RNFLT is related to age, ocular parameters and lifestyle factors. Considering these parameters in normative databases could improve the evaluation of peripapillary RNFLT. It is necessary to evaluate if a reduction of alcohol intake as well as the therapy of apnea or high homocysteine levels could positively influence RNFLT.

## Introduction

Glaucoma is a neurodegenerative disease, leading to thinning of the retinal nerve fibre layer (RNFL) which can be measured by optical coherence tomography (OCT). In order to evaluate RNFL thickness measurements, other potential influencing factors should be known and taken into account.

Acir et al. could show that iron deficiency anaemia can lead to local RNFL thinning. [1] RNFL thinning was also shown to be positively correlated with increase in serum urea and creatinine levels in patients with diabetic retinopathy. [2] A reduction of lipoprotein lipase and accumulation of visceral fat were shown to be potential factors of retinal neurodegenerative disorders that decrease RNFL thickness. [3] Acer et al. demonstrated that migraine patients without aura had decreased peripapillary RNFL thickness in temporal and nasal-superior sectors compared with control patients.[4] Ferrandez et al. [5] found a decrease in RNFL thickness in patients with severe obstructive sleep apnoea which could be confirmed by Ozge et al. [6] Also, Alzheimer disease was found to lead to significant RNFL thinning. [7]

The above mentioned factors are only a potpourri of influencing factors and disorders that might lead to thinning of RNFL thickness. However, the exact influence of ocular, cardiovascular, morphometric and lifestyle factors on peripapillary RNFL thickness remains unknown.

It can be assumed that a person’s individual RNFL thickness is influenced not only by a few but many factors. This makes the interpretation of an individual’s measured RNFL values even more difficult and puts in question the use of small databases for RNFL thickness evaluation which do not take into account all these parameters.

To date there exist no studies which investigated comprehensively many associated parameters of RNFL thickness in an epidemiologic setting. It was the aim of the current study to evaluate the relationship of ocular, cardiovascular, morphometric and lifestyle factors with global RNFL thickness in a subgroup of the Gutenberg Health Study (GHS) and to provide a formula which allows the estimation of an individual’s RNFL thickness based on these parameters.

## Material and methods

### Gutenberg Health Study

The Gutenberg Health Study (GHS) is a prospective, population-based, observational, single-center cohort study carried out in the Rhine-Main region of Western Germany (Rhineland-Palatinate). The GHS study sample is recruited from subjects aged between 35 and 74 years at the time of baseline examination (2007–2012). The sample was drawn randomly from local governmental registry offices, in which every resident is mandatorily registered, equally stratified by sex, residence (urban or rural) and for each decade of age. The present study analyses participants of the 5 year follow-up between April 2012 and December 2013.

The study protocol and study documents were approved by the local ethics committee of the Medical Chamber of Rhineland-Palatinate, Germany (reference number 837.020.07: original vote: 22 March 2007, latest update: 20 October 2015).

In accordance with the tenets of the declaration of Helsinki, written informed consent was obtained from all participants prior to entering the study.

An important feature of the GHS design is its interdisciplinary character including ophthalmological examinations, general and cardiovascular examinations, psychosomatic evaluations, laboratory tests, and biobanking for proteomic and genetic analyses. Five years after the baseline investigation, study participants are invited to participate in a follow-up visit (FU2) including the same series of investigations. All investigations are based on standard operating procedures (SOPs).

Cognitive Performance: The TOL test (Freiburg version) [8] was applied for the assessment of cognitive function. The test examines planning ability [9] and is a test of complex executive functions. The TOL performance is inked to fluid intelligence and strongly coupled with prefrontal functioning.[10]

Ophthalmological investigations include visual acuity testing and refraction with the Humphrey® Automated refractor/keratometer (HARK) 599™, slitlamp biomicroscopy (Haag-Streit BM 900^®^, Bern Switzerland), intraocular pressure measurement with a non-contact tonometer (NT 2000^TM^, Nidek Co./Japan), non-contact central corneal thickness and keratometry measurement with the Pachycam^TM^ (Oculus, Wetzlar/Germany), non-mydriatic fundus photography, and visual field testing using frequency-doubling technology perimetry with the Humphrey Matrix Perimeter (Carl Zeiss Meditec AG, Jena, Germany). [11] In April 2012 optical coherence tomography using the Spectralis OCT (Heidelberg Engineering, Heidelberg, Germany) was added to the study protocol.

### Optical coherence tomography

OCT is a non-contact, non-invasive imaging technique using light reflection in different retina levels to produce high-resolution, two-dimensional cross-sectional and 3D images.

In the current study, a circular scan was manually placed in the center of the optic disc while the eye tracking system was activated. The mean peripapillary RNFLT of the scans was estimated automatically by the software. The RNFL limits of the circle scans were automatically determined based on the software algorithms. This way, the retinal nerve fiber layer was automatically segmented in each image. These RNFL limits were then used to estimate global and sectoral values for the retinal nerve fiber layer thickness. The Spectralis OCT software, Heidelberg Eye Explorer, was used for the automatic segmentation of the RNFL and for the calculation of the RNFL thickness.

Data acquisition was adjusted for the refraction of the examined eye. No pupil dilation was performed. The scan quality ranged from 0 (no signal) to 40 (excellent) and only high-quality images (centered and well-focused optic disc with a signal strength > 15 dB) were selected for this study. All scans were quality controlled and manually checked before enrollment. Segmentations were manually re-adjusted when necessary. One scan was acquired for each eye of every subject.

### Statistical analysis

Subject demographics, age, sex, ocular, cardiovascular, morphometric and lifestyle characteristics were described by mean, standard deviation, minimum and maximum, median and quartiles for quantitative variables and by absolute and relative frequencies for categorical variables.

The association with age, sex, ocular, cardiovascular, morphometric and lifestyle factors on RNFLT was analysed using Pearson correlation coefficient and a mixed linear model with RNFLT as endpoint and the above mentioned explanatory variables as covariates. In order to account for dependence between eyes within a subject, subject was included as a random effect into the model. This analysis essentially can be interpreted as linear regression which takes dependence into account.

We then analysed the best predictive model using linear models separately for right and left eyes with stepwise backward selection and Schwarz Bayesian criterion. All independently associated parameters, determined by this procedure, were then included into a final linear mixed model with global RNFL thickness as dependent variable.

In addition, we analysed the relationship between cognitive function and RNFLT using a mixed linear model adjusted for age and sex as covariates.

Because of the explorative character of the analysis, p-values should be interpreted as a continuous measure of strength of statistical evidence.

## Results

3224 eyes of 1973 subjects (49% women) aged between 40 and 80 years were included in this study. Table 1 presents demographics of the study population and the distribution of all categorical variables. More than half of the subjects (51.9%, n = 1024) suffered from elevated blood pressure, 2.8% (n = 56) had a history of myocardial infarction and 2.6% (n = 51) reported a history of glaucoma out of whom 84% (n = 43) were treated with medication or surgery.

**Table 1:** Demographics of the study population and distribution of categorical variables.

	N	%	Missing data (N; %)
**Sex**			
male	1006	51	1, 0.1
female	966	49	
**Age decades**			
40–49	463	23.5	
50–59	604	30.6	
60–69	534	27.1	
70–80	372	18.9	
**Hypertension**	1024	51.9	4; 0.2
**History of myocardial infarction**	56	2.8	1; 0.1
**History of stroke**	32	1.6	2; 0.1
**History of coronary artery disease**	96	4.9	1; 0.1
**History of peripheral artery disease**	83	4.2	1; 0.1
**History of tinnitus**	244	12.4	3; 0.2
**History of migraine**	534	27.1	2; 0.1
**History of sleep apnea**	161	8.2	1; 0.1
**Ever smoked**	1052	53.3	4; 0.2
**History of diabetes**	146	7.4	
**Alcohol intake**	1143	57.9	
≥ 10g/d (women) / ≥ 20g/d (men)	509	25.8	
**History of glaucoma**	51	2.6	
**Glaucoma therapy (medication or surgery)**	43	2.2	
**Pseudophakia**	101	5.1	
**Eye surgery**	125	6.3	

N = number of cases

Table 2 presents further demographics of the study population and the distribution of quantitative variables including data for global retinal nerve fiber layer thickness (RNFLT).

**Table 2:** Demographics of the study population and distribution of quantitative variables.

	N	N miss	mean	SD	Min	Q1	Median	Q3	Max
**Systemic parameters (persons)**									
**Body weight** **[kg]**	1973	0	79.8	15.9	45.6	68.5	78.7	89.1	162.2
**Body length [cm]**	1973	0	170.4	9.3	137.0	163.4	170.2	177.0	198.5
**BMI**	1973	0	27.44	4.74	17.17	24.21	26.82	29.93	52.27
**Cholesterol [mg/dL]**	1968	5	222.7	42.0	70	194	220	248	419
**HbA1c [%]**	1965	8	5.66	0.58	4.5	5.3	5.6	5.9	11.6
**Glucose [mg/dL]**	1969	4	92.6	16.0	49	84	90	97	290
**Homocysteine**	1960	13	11.9	4.6	4.6	9.3	11.1	13.6	73.0
**Ocular parameters (eyes)**									
**Spherical equivalent**	3217	7	-0.29	2.14	-12.5	-1.13	-0.13	0.88	8.00
**Intraocular pressure**	3156	68	14.89	2.91	7.00	13.00	14.7	16.7	26.7
**Axial length**	2901	323	23.62	1.15	18.29	22.92	23.57	24.27	28.48
**Central corneal thickness**	2890	334	548.5	34.5	405	525	549	571	773
**RNFLT global**	3224	0	96.1	10.4	48	90	96	103	207

N = number of cases; N miss = number of missing cases, SD = standard deviation, Min = minimum, Q1 = quantile 1, Q3 = quantile 3, Max = maximum, BMI = body mass index, RNFLT = retinal nerve fiber layer thickness.

Pearson correlation coefficients were calculated in order to analyze univariate associations between RNFLT and age, sex, ocular, cardiovascular, morphometric and lifestyle factors. Highest absolute correlations were found for axial length (r = -0.27), spherical equivalent (r = 0.24), and known glaucoma (r = -0.15) (p<0.0001, respectively). Other correlations with RNFLT were found for age (r = -0.12, p<0.0001), sex (r = 0.05, p = 0.0023), intraocular pressure (r = -0.07, p = 0.0002), hypertension (r = -0.05, p = 0.002) and systolic blood pressure (r = -0.07, p = 0.0002), previous eye surgery (r = -0.04, p = 0.03), cholesterol (-0.04, p = 0.03), homocysteine (r = -0.08, p<0.0001), history of coronary artery disease (r = -0.05, p = 0.01), history of myocardial infarction (r = -0.05, p = 0.01), apnoea (r = -0.04, p = 0.01), diabetes (r = -0.04, p = 0.03) and alcohol intake (r = -0.05), p<0.05, respectively. High alcohol intake (>10g/day in women and >20g/day in men) was correlated more strongly (r = -0.08, p<0.0001) than lower alcohol intake (<10 / 20g/day) (r = -0.05, p = 0.01).

Body size, body weight, BMI, diastolic blood pressure, blood glucose, HbA1c, history of apoplexy, peripheral artery disease, tinnitus, migraine as well as nicotine intake did not show correlation with RNFLT beyond chance. Neither did central corneal thickness, or pseudophakia.

In the multivariable model age, sex, spherical equivalent, axial length, history of glaucoma, history of tinnitus and high alcohol intake remained independently associated, while all other parameters were not related to global retinal nerve fibre layer thickness (Table 3).

**Table 3:** Multivariable linear regression model to estimate associations with retinal nerve fiber layer thickness in the Gutenberg Health Study.

Effect	Estimate	p-value
**Intercept:**		
Male, 40–49 years	125.74	
Male, 50–59 years	124.63	
Male, 60–69 years	121.83	
Male 70–80 years	121.18	
Female, 40–49 years	127.73	
Female, 50–59 years	124.92	
Female, 60–69 years	123.85	
Female 70–80 years	119.59	
**Spherical equivalent (per dioptre)**	1.33	**<0.0001**
**Intraocular pressure (per mm Hg)**	-0.08	0.28
**Axial length (per mm)**	-1.29	**<0.0001**
**Central corneal thickness (per μm)**	0.0001	0.99
**Pseudophakia (yes)**	-0.31	0.90
**Eye surgery (yes)**	2.24	0.31
**Self-reported glaucoma (yes)**	-7.69	**<0.0001**
**Systolic blood pressure (mm Hg)**	0.0006	0.98
**Diastolic blood pressure (mm Hg)**	-0.02	0.65
**History of myocardial infarction**	-1.04	0.53
**History of stroke**	-0.35	0.85
**History of coronary artery disease**	-0.07	0.96
**History of peripheral artery disease**	-0.70	0.54
**History of tinnitus**	1.38	**0.04**
**History of migraine**	0.07	0.90
**History of apnoea**	-1.70	0.05
**Body length (per cm)**	0.02	0.52
**Body weight (per kg)**	0.03	0.07
**Cholesterol (per mg/dl)**	-0.0008	0.89
**HbA1c (per mg/dl)**	-0.01	0.98
**Glucose (per mg/dl)**	0.006	0.73
**Homocysteine (per mg/dl)**	-0.10	0.05
**ever smoked (yes)**	-0.45	0.34
**Diabetes (yes)**	-1.28	0.20
**Alcohol (yes)**	0.12	0.83
**Alcohol** **>10g/day (female) / >20g/day (male)**	-1.46	**0.02**

After stepwise backward elimination, age, spherical equivalent, axial length and history of glaucoma remained in the model best predicting global retinal nerve fibre layer thickness (Table 4).

**Table 4:** Best fitting model to predict global retinal nerve fiber layer thickness in the Gutenberg Health Study.

Multivariable mixed linear regression model		Estimate (in μm)	Standarderror	Pr > |t|
**Reference: 70–80 years:**		121	5.2	
** Age 40–49 years**	40–49	6.6	0.7	< .0001
** Age 50–59 years**	50–59	4.3	0.7	< .0001
** Age 60–69 years**	60–69	2.1	0.7	0.0018
**Spherical equivalent (per dpt)**		1.3	0.1	< .0001
**Axial length (per mm)**		-1.2	0.2	< .0001
**Self-reported glaucoma (yes)**		-7.4	1.4	< .0001

Included parameters were determined using stepwise backward selection to find the best model for prediction (based on Schwarz Bayesian criterion) of global retinal nerve fiber layer thickness.

Regarding cognitive function, we did not find an association between tower of London score and RNFLT (p = 0.50), adjusted for age and sex.

## Discussion

As part of the population-based Gutenberg Health Study, we demonstrate that peripapillary RNFL thickness is linked to aging and biometric parameters, namely refraction and axial length. PRNFL thickness is lower in persons with glaucoma, and interestingly in persons with high alcohol intake.

More recently, several groups evaluated influencing factors on peripapillary RNFL thickness using different OCT devices. Leung et al. showed that high myopia is linked to thinner pRNFL.[12] Oner et al. found a relationship between myopic refraction and thinner and average pRNFL thickness using the RTVue OCT (Optovue, Fremont, CA). In addition, longer axial length was linked to thinner pRNFL measurements and the authors suggest including axial length as parameter in normative databases.[13] Lee et al. evaluated the intra-individual influence of refractive power and compared the influence of different soft contact lenses on pRNFL measurement.[14] They reported a lower pRNFL thickness with increasing myopia and vice versa. In a small cohort, Schuster et al. reported an association between thinner pRFNL and myopia in otherwise healthy eyes using Topcon 3D-OCT 2000 (Tokyo, Japan).[15] Our study confirms these findings showing an independent association of thinner pRNFL with myopia and longer axial length using Spectralis-OCT. Whether these findings are due to ocular magnification, as discussed by Khan et al. using a Cirrus HD OCT-system [16], is still controversial. In contrast to the Cirrus HD OCT-system, the Spectralis-OCT system do not report peripapillary retinal nerve fiber layer thickness measured with with a fixed absolute scan diameter (i.e. 3.46mm), but on a 12° diameter.

There are few studies evaluating the influence of systemic factors on pRNFL thickness. Khawaja et al showed an association of thinner pRNFL thickness as measured with a GDxVCC device with a higher body mass index.[17] A Chinese population-based study in non-glaucomatous subjects showed age and axial length as associated factors for peripapillary RNFL thickness determined with SD-OCT, while systemic parameters (arterial blood pressure, body mass index, HDL and LDL cholesterol, triglycerides) except age did not have an impact.[18] Similar, Schuster et al. did not find any classical systemic cardiovascular risk factors to be associated with pRNFL thickness in apparently healthy subjects after correction for ocular parameters.[15] Our study confirms these findings on basis of a population-based study setup and therefore risk of selection bias is low.

Interestingly, we found an association of thinner peripapillary RNFL thickness and risky alcohol intake according to WHO definition (≥ 10g/d (women) / ≥ 20g/d (men)). Similarly, Khawaja et al. found a univariate association between alcohol intake and peripapillary RNFL thickness as assessed with GDxVCC device, but could not confirm it after adjustment for covariates.[17] The intake of alcohol seems not only to have an impact on neuronal degeneration, but also on neuronal development. A recent study of patients with fetal alcohol syndrome showed that these have a decreased peripapillary RNFL thickness.[19] These patients did not show temporal RNFL thinning, in contrast to case reports of tobacco-alcohol-induced toxic optic neuropathy.[20]

Apart from technical examinations, fundus examination of RNFL maintained that several systemic factors such as older age, male sex, hyperglycemia and dyslipidemia, had an influence on RNFL visibility. [21] High arterial blood pressure and higher concentration of low-density lipoproteins were associated with localized RNFL defects.[21]

We did not find an association between cognitive function and RNFL thickness which is in contrast to several other studies. Oktem et al. found an association between cognitive functioning measured with mini mental state examination (MMSE) test and RNFL thickness,[22] as did Khawaja et al. using the short form of MMSE.[23] These tests examine different characteristics of cognitive functioning compared to the tower of London test as a planning ability test. A recent meta-analysis [24] analyzed the relationship of RNFL thickness with Alzheimer’s disease and mild cognitive impairment and found substantial heterogeneity between the studies due to methodology. High-quality studies indicated that global RNFL thickness was thinner in subjects with Alzheimer’s disease or mild cognitive impairment compared to normal controls. Apparently, thinning of the RNFL is linked to neurodegenerative processes which are only marginally represented in the population-based sample.

Although our study population was randomly drawn from the population, all participants underwent standardized ophthalmic and cardiovascular examinations and reports on a relatively large sample size, it has several limitations. First, we did not analyze all participants of the cohort, but a subsample, as sufficient image quality was not always available. Therefore, we performed an item-nonresponder analysis and found to have included a comparable sample with slightly younger age. In addition, our population-based study has a response rate of 84% and therefore our underlying cohort might not perfectly reflect the general population with an age range of 40 to 79 years. We did not measure the size of the optic disc and therefore cannot control for this parameter in our analysis. In contrast to other devices, Spectralis-OCT performs a peripapillary circle with a diameter of 12°, which is less influenced by the optical biometry compared to circles with an absolute diameter (i.e. 3.4 mm).

In conclusion, our study analyzed associated factors with RNFL thickness readings using spectral-domain OCT. Our analysis found age, refraction and axial length as major influencing factors, while cardiovascular parameters (body weight, arterial blood pressure, biochemical parameters, history of stroke or myocardial infarction) did not reach significance. High alcohol intake was associated with thinner RNFL. A prospective study comparing patients with and without alcohol use is needed to show the impact of alcohol on RNFLT. A marginal association with history of tinnitus was found, which further studies need to support for the interpretation of RNFL thickness readings. Current data indicates that clinical evaluation of RNFL thickness is independent of cardiovascular parameters, but refraction and axial length has to be kept in mind when interpreting RNFL data.
